# SARS-CoV-2 Vaccination Coverage in Italian Children with Celiac Disease

**DOI:** 10.3390/jcm13195851

**Published:** 2024-09-30

**Authors:** Carlotta Pepino, Federica Malerba, Valentina Biagioli, Talia D’Ambrosio, Noemi Zampatti, Francesca Canzoneri, Jacopo Ferro, Marco Crocco

**Affiliations:** 1 Department of Neuroscience, Rehabilitation, Ophthalmology, Genetics, Child and Maternal Health, University of Genova, 16100 Genova, Italy; carlottapepino@gaslini.org (C.P.); federicamalerba@gaslini.org (F.M.); valentina.biagioli@edu.unige.it (V.B.); noemi.zampatti@gmail.com (N.Z.); francesca_canzoneri@libero.it (F.C.); 2 Paediatric Clinic, University of Ferrara, 44124 Ferrara, Italy; taliadambrosio@gmail.com; 3 Pathology Unit, IRCCS Istituto Giannina Gaslini, 16147 Genova, Italy; jacopoferro@gaslini.org; 4 Pediatric Gastroenterology and Endoscopy Unit, IRCCS Istituto Giannina Gaslini, 16147 Genova, Italy

**Keywords:** celiac disease, COVID-19, vaccination, SARS-CoV-2, autoimmunity, gluten, vaccine hesitancy

## Abstract

**Background**: Celiac disease (CD) is the most common multisystemic autoimmune disorder affecting the pediatric population. However, little data is available regarding SARS-CoV-2 vaccination coverage in pediatric patients with CD. This study aims to evaluate the adherence to national recommendations for SARS-CoV-2 vaccination in children and adolescents with CD and its variation over time. **Methods**: We retrospectively analyzed medical charts and electronic registry records of SARS-CoV-2 vaccination of patients aged 0–19 years diagnosed with CD in a tertiary center. The vaccination coverage was evaluated according to age groups (young children, children, and adolescents), considering the patients’ eligibility for vaccination at different times. **Results**: Among the 172 patients enrolled, 44.8% received at least one dose of the SARS-CoV-2 vaccine, showing no significant differences compared to the Italian population of similar age. Vaccination coverage demonstrated a progressive reduction after an initial peak (up to 65.5% in December 2021) concomitant with a gradual extension of vaccinable eligibility and falling SARS-CoV-2 infections. Histological diagnosis and the presence of other associated autoimmune diseases were associated with higher levels of adherence to vaccination. **Conclusions**: Adherence to the SARS-CoV-2 vaccination in young Italian children with CD was very low, while it was better in adolescents and patients with other associated autoimmune diseases. Vaccine hesitancy remains a concern, particularly among those diagnosed using the biopsy-sparing approach. Hesitancy increased during the pandemic period, suggesting the need for ongoing efforts to improve adherence to SARS-CoV-2 vaccination recommendations.

## 1. Introduction

Celiac disease (CD) is a multisystemic autoimmune disorder characterized by enteropathy in response to intestinal exposure to gluten in genetically predisposed individuals [1]. Patients with CD may develop various complications, including other autoimmune diseases, cancers, and low quality of life [2,3,4,5]. In Western countries, the incidence of CD has been increasing over the last several decades [6]. Awareness of the disease has increased, but CD remains markedly underdiagnosed, and the diagnostic delay is relevant [7]. In a recent Italian multicenter, cross-sectional study, the overall prevalence of CD in Italy was 1.65% (95% coefficient interval, 1.34–2.01) [8]. Various environmental factors, including certain viruses, constitute important triggers in the pathogenesis of CD [9]. Epidemiological studies have shown that viral infections may induce autoimmunity through multiple mechanisms, including molecular mimicry, epitope spreading, bystander activation, and immortalizing infected B cells [10]. The effect of SARS-CoV-2 infection on the onset of CD in children is under investigation, with conflicting data reported [11,12,13]. Patients with CD have a similar likelihood of contracting COVID-19 when compared with healthy controls [14] and are not at increased risk of hospitalization or death [15,16].

The COVID-19 pandemic, caused by the SARS-CoV-2 virus, has had a profound impact globally, including widespread morbidity, mortality, and significant disruption of children’s daily lives. Italy was the first European country to face the COVID-19 outbreak. The SARS-CoV-2 vaccine rapidly emerged as a critical tool in controlling the spread of the virus and reducing the severity of COVID-19. In Italy, vaccination is offered to people based on scientific consensus, European recommendations, and local epidemiology policies, including cultural values and principles of equity, reciprocity, legitimacy, protection, and promotion of public health and well-being, always considering factors such as the risk of disease and vaccine adverse effects [17]. In many countries, including Italy, vaccination campaigns were first actively pursued to achieve the highest levels of widespread immunity among the elderly and other high-risk groups, such as those with chronic diseases. In March 2021, the Ministry of Health issued a decree adopting a new National Strategic Plan for preventing SARS-CoV-2 infections and implementing a national vaccination campaign [18]. This plan, based on the consideration mentioned above, established the characteristics that determined the priority for vaccination. Thanks to effective collaboration and rapidly implemented legislation, a comprehensive network of 1700 vaccination sites and a dedicated vaccine distribution network was established to implement SARS-CoV-2 vaccination. This network involved a substantial number of healthcare providers, including 44,000 general practitioners, 60,000 dentists, and 23,000 resident doctors. Moreover, during the various “waves” of infections, a series of measures, such as social distancing, were adopted to limit the spread of SARS-CoV-2.

On 1 December 2021, the Technical-Scientific Committee of AIFA, in accordance with the European Medicines Agency (EMA), approved the extension of the indication for the Comirnaty (BNT162b2, BioNTech/Pfizer) vaccine in a specific formulation of 10 mcg/dose for children aged 5 to 11. On 16 November 2022, vaccination was further extended to children aged six months to four years without underlying health conditions, based on the request of a parent or legal guardian. The Comirnaty vaccine was the first to be authorized and, for a considerable time, the only one available for use in the pediatric population in Italy. The first Comirnaty vaccine was developed using the genetic sequence of the virus circulating at the beginning of the pandemic (Wuhan/original/ancestral variant). Subsequently, the following updated formulations targeting circulating variants were developed and authorized: Comirnaty Original/Omicron BA.1 (bivalent), authorized on 21 January 2022; Comirnaty Original/Omicron BA.4/BA.5 (bivalent), authorized on 1 September 2022; Comirnaty Original/Omicron XBB.1 (bivalent), authorized on 18 January 2023 [19]. The development of safe and effective SARS-CoV-2 vaccines was crucial to reduce COVID-19 mortality rates and, eventually, the social restrictions [20,21,22]. Thanks to the SARS-CoV-2 mass immunization campaigns that started on 27 December 2020, Italy reached one of the highest levels of SARS-CoV-2 vaccination coverage in Europe in the first months of the vaccination plan. However, in many Western countries, including Italy, the phenomenon of “vaccine hesitancy” grew alongside the availability of vaccination services, and despite evidence of their efficacy [23,24,25], this phenomenon also extended to the pediatric population.

While generally experiencing milder forms of COVID-19 than adults, children remain an important group for vaccination to reduce the long-term effects of COVID-19 and rare cases of severe disease. It also limits transmission to potentially vulnerable family members. Among the pediatric population, children with chronic autoimmune diseases, such as CD, may present an increased risk of vaccine hesitancy related to recent experience of illness and a fear of vaccine-related flare-ups of their disease. In Italy, which has a high prevalence of CD, little data are available on SARS-CoV-2 vaccination coverage in the pediatric population with CD. Assessing the vaccination coverage among children with this condition is crucial to understanding the rate of hesitancy. It provides insights into the effectiveness of public health campaigns, helping ensure that these children are not left vulnerable to COVID-19. This aimed to assess SARS-CoV-2 vaccination and its variation over time in children and adolescents suffering from CD residents in the province of Genova.

## 2. Materials and Methods

We retrospectively analyzed data on SARS-CoV-2 vaccination in patients diagnosed with CD from 2018 to 2022 at our regional center for CD, “Giannina Gaslini” Children Hospital. All children and adolescents (<20 years old) resident in the province of Genoa were eligible. Patients vaccinated before CD diagnosis, not diagnosed according to ESPGHAN guidelines [26,27], or not registered in the regional health system were excluded. Data on SARS-CoV-2 vaccination (number and date of vaccination) were taken from electronic charts in January 2024; all charts were verified through comparison with the electronic vaccination registry of the regional health system. The vaccination was evaluated according to age groups, considering the patients’ eligibility for vaccination at different points in time (that is, considering the authorization for vaccination use in different age groups issued by national and European authorities). We evaluated vaccination coverage the day before authorization for the “new” age group to identify vaccine hesitancy in different age groups better. In particular, we evaluated the coverage of vaccination on these specific dates: 28 May 2021, corresponding to the last day before authorization was extended to adolescents 12–15 years old; 1 December 2021, corresponding to the last day before authorization was extended to children 5–11 years old; 24 October 2022, corresponding to the last day before authorization was extended to young children (<5 years old and >6 months); 1 January 2024, last day of evaluation.

The following clinical data were extracted from electronic charts and analyzed: gender, age at diagnosis, date of CD diagnosis, type of diagnosis (histological or biopsy-sparing approach), reasons for screening, gastrointestinal and extra-intestinal symptoms at diagnosis, eventual comorbidity, selective immunoglobulin (Ig) A deficiency, and family history of CD.

Data was summarized using means and standard deviations (SD) for continuous variables and absolute frequencies and percentages for categorical variables. The difference between the two means was calculated using a Student’s *t*-test. The Chi-square test was used to compare the coverage of SARS-CoV-2 vaccination among patients with CD at our regional center with that of the Italian general population under 20. Additionally, the test with Yates’ correction was used to evaluate differences between patients who received a clinical diagnosis of CD and a histological diagnosis of CD or between other clinical variables. A *p*-value of ≤0.05 was considered statistically significant for all two-tailed tests performed. Prisma GraphPad Software version 8.3 (Dotmatics, Boston, MA, USA) was used for all the analysis.

The study was conducted in accordance with the Declaration of Helsinki, and the protocol was approved by the Regional Ethics Committee (Liguria: 133/2024—DB id 13773, 29 April 2024). Written assent and consent to participate were obtained from all participants (and parents or legal guardians) in this study.

## 3. Results

**Figure 1:**
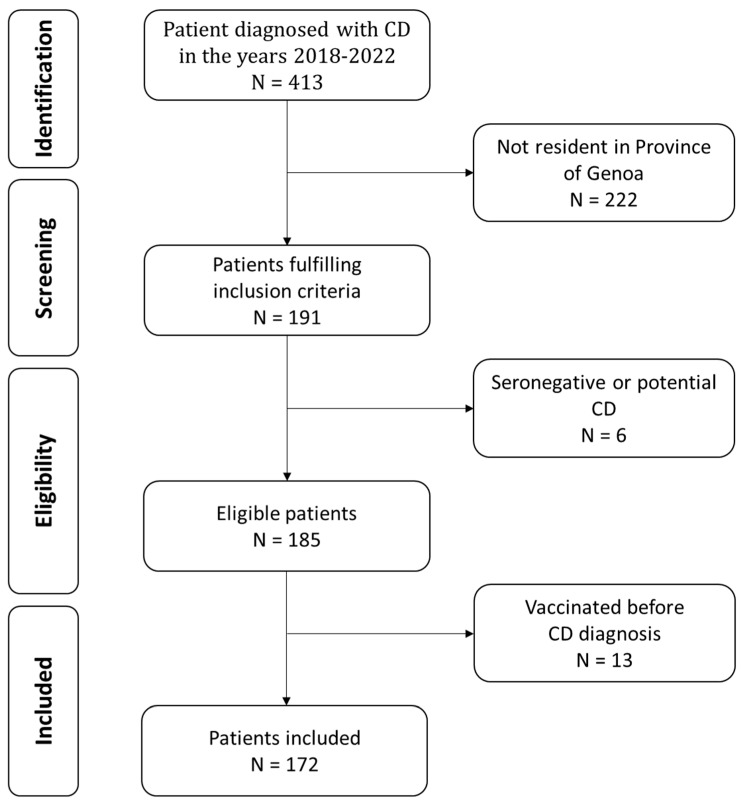
Flowchart of patient inclusion.

The total number of children diagnosed with CD at our hospital from 2018 to 2022 was 413; 191 of them were residents in the province of Genoa; six were excluded because they had not been definitively identified as CD during the period studied. We also excluded 13 patients because they had been vaccinated before their CD diagnosis (Figure 1). This study includes 115 females (66.9%) and 57 males (33.1%). Of the 172 total patients enrolled, 75 (43.6%) were diagnosed through biopsy, and 97 (56.4%) were diagnosed using clinical ESPGHAN criteria. The mean age at diagnosis of CD was 8.1 ± 4.5.

The reasons for screening for CD were categorized into four main groups. The primary reason for the screening was the presence of symptoms (121 participants, 70.3%), followed by those due to a family history of CD for the presence of first-degree relatives with CD (29 patients, 16.9%), incidental findings (20 participants, 11.6%), and those belonging to a risk group (13 participants, 7.6%). Some patients (9, 5.3%) had multiple reasons listed, indicating overlaps in screening criteria. At the onset of CD, out of the 172 participants, 82 (47.7%) presented gastrointestinal symptoms (47 recurrent abdominal pain, 21 diarrhea, 20 constipation, eight intestinal flatulence, seven weight loss, and seven vomiting); 95 (55.2%) presented extra-gastrointestinal symptoms (38 slow growth rate, 31 anemia, 23 fatigue, ten headache, six loss of appetite, two dermatitis, one arthralgias, and one recurrent oral aphthosis); two (1.2%) clinical presentation compatible with celiac crises. Chromosomal disorders were diagnosed in five (2.9%) patients (one Turner syndrome, one DiGeorge syndrome, and three complex chromosomal abnormalities), and 13 patients (7.6%) suffered from other autoimmune disorders (10 type 1 diabetes mellitus, two alopecia, one vitiligo, and one systemic idiopathic arthritis) at the time of screening. Only six (3.5%) were identified with IgA deficiency; the clinical presentation in this group was one celiac crisis, two presented typical symptoms, and three any symptoms (2 screened due to a first-degree family history of CD).

All patients in the study received the Comirnaty (BNT162b2, BioNTech, Mainz, RP, Germany/Pfizer New York City, NY, USA) vaccine, as recommended by the Italian Ministry of Health [19]. Comirnaty is indicated for active immunization to prevent COVID-19 caused by SARS-CoV-2 in individuals aged six months and older. The vaccine was administered intramuscularly, following dilution, preferably in the deltoid muscle of the arm, as part of a primary series of three doses. The second dose was given three weeks after the first, and the third dose was administered at least eight weeks after the second. For individuals aged 12 and older, a dose of 30 micrograms (mcg) was administered, while those aged 5 to 11 years received a 10 mcg dose. In the age group of six months to four years, a 3 mcg dose was used. If a child transitioned to an older age group between doses during the vaccination course, they continued to receive the dose appropriate to their previous age group to complete the series. In our study, verifying the type of Comirnaty vaccine was impossible.

**Figure 2:**
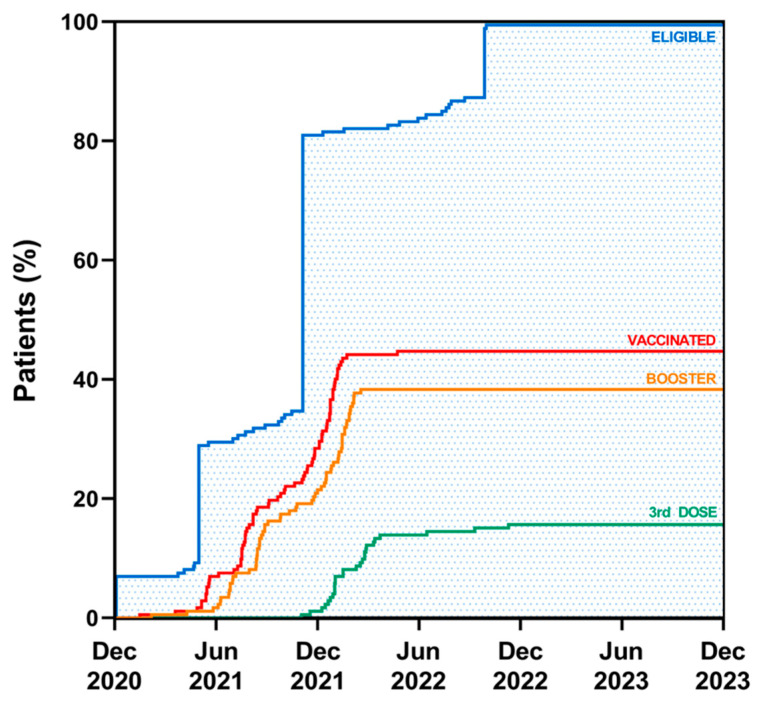
SARS-CoV-2 vaccination coverage in pediatric patients with CD at different time points.

**Figure 3:**
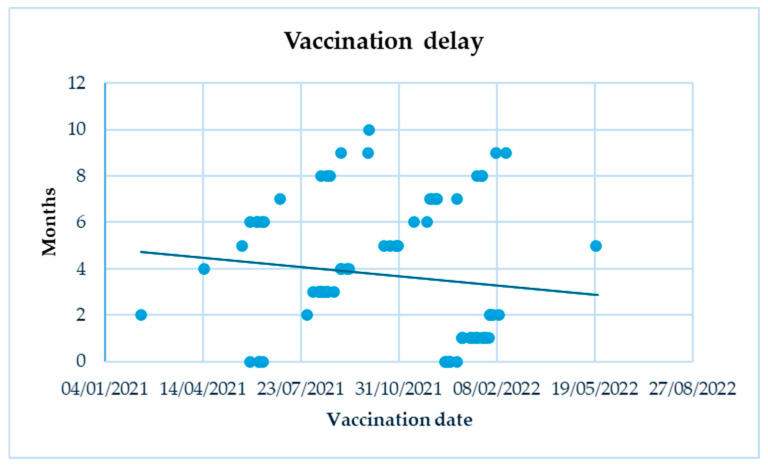
Vaccination delay over time.

Concerning the number of doses effectuated, 14.3% (11 patients) received only one dose, 50.6% (39 patients) received a booster dose, and 35.1% (27 patients) received a 3rd vaccination. The mean age at first vaccination was 12.5 ± 3.8 years old. Patients who had not received the vaccination by 1 January 2024 were, on average, diagnosed with CD more recently than those who had been vaccinated (3.3 ± 1.5 versus 4.1 ± 1.1, *p* < 0.0005). There were no statistically significant differences in vaccination rates between the two genders (47.8% in females versus 38.6% in males). The prevalence of vaccination by age range was 0% in very young children (<5 years old), 35% (27/77) in children, and 65% (50/77) in adolescents. The overall coverage of SARS-CoV-2 vaccination in children and adolescents with CD was 44.8% (77 patients) on 1 January 2024, with a significant (*p* < 0.0005) higher prevalence in patients with histological diagnosis (61.8%, 47/76) compared to those diagnosed with ESPGHAN biopsy-sparing approach (31.3%, 30/96). There were no significant differences in the percentage of vaccinated individuals between patients with and without symptoms at the onset of CD. Among patients with other autoimmune diseases, the prevalence of vaccination was 61.5% (8/13). Evaluating the level of take-up at various points of time (related to the points of authorization of the vaccination) (Figure 2), that is, on the last day before authorization was extended to adolescents 12–15 years old (28 May 2021), 26 patients could have been vaccinated (due to their age being at least 16 years old), but only three (11.5%) had received a dose. However, 12 more patients (of these 26) received vaccinations in the following period (that is, in the period in which vaccine authorization was extended to adolescents 12–15 years old). On 1 December 2021, a total of 40 adolescents (65.5% of the total 61 vaccinable aged at least 12 years) had been vaccinated. In the following period (1 December 2021–24 October 2022), 37 patients received vaccination (10 of whom could have been vaccinated in the previous period); on 24 October 2022, 77 patients (51%) of the 151 that could have been vaccinated, i.e., aged at least five years, had received a dose. No patients received vaccination after 24 October 2022. Therefore, on the last day of evaluation (1 January 2024), 77 patients (44.8%) of the 172 that could have been vaccinated, i.e., aged at least six months, had been vaccinated. The time gap between the authorization date for a specific age group and the actual vaccination was 3.7 ± 2.9 months, showing a decreasing trend over time (Figure 3).

## 4. Discussion

This is the first study on SARS-CoV-2 vaccination coverage in children and adolescents affected by CD. Our findings reveal a relatively low overall vaccination coverage of 44.8% among our cohort with CD, with significant differences appearing to be associated with age group and the method of diagnosis.

Our study showed that younger age was associated with high familial hesitancy to vaccination while having another autoimmune disorder, and being older (children/adolescents) was associated with a greater propensity for parents to vaccinate their children. Less than half of families chose to vaccinate their children with CD. However, compared with the overall Italian population [28], our results demonstrate a slightly higher take-up of SARS-CoV-2 vaccination in children and adolescents with CD (39.6% in Italian general population < 20 years old), although not significant (*p* = 0.16).

Evidence on outcomes of COVID-19 infection revealed an increased association of severe COVID-19 in younger children (those aged 2–11 years) compared with older children (those aged 12–18) and in patients with one or more chronic conditions versus those without [29]. Nevertheless, in our study, the prevalence of vaccinated patients appears inversely related to age. The younger the patient, the lower the likelihood of vaccination, with no children aged less than five years old having been vaccinated. The analysis of different age groups showed that after an initial peak of vaccination, a progressive reduction in vaccination coverage was concomitant with an increase in the vaccinable (younger) age. These results are in line with Italian data provided by the Ministry of Health, updated to 24 September 2023 [28], which show that in our region (Liguria), coverage of 79.2% of adolescents (12–19 years) and 28.8% of the pediatric population (5–11 years old) with very few children under five years having been vaccinated. However, the cumulative uptake of the primary course by age group in European Economic Agreement (EEA) countries shows that, in the age group < 18 years, Italy is the country with the highest prevalence of vaccination [30]. This (age) trend is confirmed by Miraglia del Giudice et al. [24], who showed that only one-third (38.8%) of the parents would vaccinate their children against SARS-CoV-2, and those whose child was older were more likely to express a greater willingness to vaccinate their child (OR = 1.18; 95% CI = 1.05–1.32). This discrepancy may reflect broader trends observed in the general population, where vaccine hesitancy tends to be higher among parents of younger children due to concerns about vaccine safety, particularly with newer vaccines such as those for SARS-CoV-2 with messenger RNA (mRNA) technology. The COVID-19 vaccines have been developed using various technologies, resulting in different types available for public use. The mRNA vaccines, such as Comirnaty (Pfizer-BioNTech) and Spikevax (Moderna), use mRNA to instruct cells to produce a harmless spike protein found on the surface of the virus, triggering an immune response. The Comirnaty vaccine was the first to be authorized, and it remains the most widely used in Italy (particularly in children) [19]. After a period of popularity for these vaccines, linked to the advice in 2021 of increased new rare adverse effects (i.e., a combination of thrombosis and thrombocytopenia) following vaccination with viral vector vaccines (Johnson & Johnson, Janssen and AstraZeneca, and Vaxzevria), the new technology became a source of hesitation for families in different countries. Vaccine booster shots face even greater hesitancy compared to the initial two doses of COVID-19 vaccines [31,32]. A survey conducted online between July and August 2023 among a sample of 4303 French adults demonstrated that 62% of participants reported still having doubts about the long-term side effects of mRNA vaccines, and 31% agreed that mRNA vaccines are not real vaccines but rather gene therapies [33].

The lower uptake in younger children could potentially be attributed to the later approval of vaccines for these age groups, leading to a shorter timeframe for vaccination compared to older children and adolescents. However, we also report a change in SARS-CoV-2 vaccine hesitancy over time, with a lower rate of children and adolescents vaccinated even several months after the authorization of the vaccination for that age group. This trend may be explained by the increasing hesitancy and strong convictions of parents who refuse vaccination due to personal ideas often acquired and reinforced through social media.

A significant finding of this study is the higher vaccination coverage among patients diagnosed via biopsy compared to those diagnosed using the ESPGHAN biopsy-sparing approach. This difference may indicate that parents and caregivers of children who underwent a biopsy, a more invasive procedure, might perceive the severity of the disease differently, potentially leading to greater trust in medical interventions, including vaccination. Similarly, a higher prevalence of vaccination was also confirmed in children affected by immunodeficiencies and onco-hematologic diseases [34,35]. In contrast, the non-invasive nature of the ESPGHAN criteria might be associated with a lower perception of the disease, thereby influencing vaccine hesitancy. This finding highlights the need for targeted communication strategies to address vaccine hesitancy, particularly in patients diagnosed younger and through non-invasive methods.

In a web-based survey of parents, the most frequent causes of SARS-CoV-2 vaccine hesitancy in the USA were fear of vaccine side effects (61.5%) and doubts about vaccine safety (48.96%) [36]. Scaramuzza et al. [37] found that 20% of parents of children affected by type 1 diabetes are against vaccination, mainly due to being worried about side effects (especially long-term) or to the perception of a low risk of SARS-CoV-2 infection and complications in children; these results were confirmed by Campagnani et al. [38]. Other parents are concerned about the rapid vaccine production process, as found by Pourrazavi et al. [39]. As expected, parents are more in favor of vaccination if their children have been hospitalized for COVID-19 in the past [40]. A survey conducted in Italy in February 2021 indicated that 25.2% of the 103 adults with CD were hesitant (5% refused) about SARS-CoV-2 vaccination, and almost one-quarter of them reported that their hesitancy was influenced by CD [41].

Vaccine hesitancy, defined by WHO as “the reluctance or refusal to vaccinate despite the availability of vaccines”, is a complex, multifaceted behavior that may reduce the take-up and efficacy of vaccination campaigns; it was sufficiently well-known prior to the pandemic that the WHO defined it as one of the ten threats to global health [42,43]. This phenomenon is particularly pronounced in parents with young children and has become much more difficult to address since the COVID-19 pandemic, in line with the growth of social media. The reasons why parents choose not to vaccinate their children are complex, varying across time, place, and type of vaccines [44]. The Strategic Advisory Group of Experts (SAGE) on immunization identified complacency, inconvenience in accessing vaccines, and lack of confidence as the most important factors in hesitancy. Acceptance of vaccination is an outcome behavior involving children, caregivers, and physicians resulting from a complex decision-making process built on three main characteristics: confidence, complacency, and convenience [25]. Confidence in vaccination is defined as trust in the effectiveness and safety of vaccines and the healthcare system. While mRNA vaccines are more efficacious than conventional vaccines, a recent study found that the novelty of the mRNA vaccine technology reduces (14.2%) the probability of vaccine acceptance [45]. Vaccination complacency arises when the perceived risks of vaccine-preventable diseases are low, and vaccination is not viewed as a necessary preventive measure. Complacency about a specific vaccine or vaccination, in general, can be influenced by various factors, including the presence of other diseases, as in our population with other autoimmune diseases in which the prevalence of vaccinated people is high. Paradoxically, the success of immunization programs can lead to complacency and, eventually, vaccine hesitancy, as individuals may perceive the risk of vaccination as greater than the risk of the “now” rarer disease. This could explain the reduction in vaccination rates in our population over time. Vaccination convenience is crucial, encompassing factors such as geographical accessibility as well as physical and economic availability. The quality and timing of the services can influence the decision to get vaccinated and may contribute to some hesitant families to vaccinate their children. In the first period under examination, the health system made an extraordinary effort to ensure rapid access to vaccination, but over time, the system returned to the ordinary regime by reducing the number of vaccination points, reducing the accessibility.

This study underscores the importance of understanding vaccine hesitancy in the context of chronic autoimmune diseases like CD. Previous research has shown that individuals with autoimmune conditions often have concerns about vaccine safety, fearing that vaccination might trigger a flare-up of their disease [24]. Although current evidence does not support an increased risk of adverse outcomes following COVID-19 vaccination in patients with CD, these fears may still influence decision-making by parents and caregivers. We believe that this data may be useful for revising behavioral guidelines on SARS-CoV-2 vaccination, pointing out the need for efforts by policymakers to improve recommendations for children and adolescents with chronic diseases. Healthcare providers should prioritize clear and evidence-based communication regarding the safety and benefits of vaccination for children with CD. The low adherence reported in children with chronic disease underlines the need for stronger engagement of specialists in preventive activities by recording chronic non-immunized patients and actively offering the vaccines, as well as considering the possibility that patients receive care from multiple subspecialists (e.g., patients with CD and diabetes type 1) and receive little or no care from primary medical care providers. However, several barriers have been reported by specialists to discussing vaccination with these patients, such as a lack of time, an ineffective “reminder” system, and the vaccine organization system [46].

This study has several limitations that should be considered when interpreting the results. First, a retrospective approach could have led to us missing some data, particularly SARS-CoV-2 infections that may explain some vaccination delays. However, thanks to cross-checking electronic charts and health registry records for vaccination, the data on take-up should be highly reliable. Second, this study was conducted in a single geographical region, which may limit the generalizability of the findings to other areas with different demographic and healthcare characteristics; a recent study also shows considerable regional variations (up to 30%) in COVID-19 vaccine uptake in the same country [47]. Third, this study analyzes vaccination coverage across different pediatric age groups; however, children and adolescents are in distinct situations, characterized by differing immune responses (vaccine and SARS-CoV-2), varying risks of contracting COVID-19, vaccination with different Comirnaty (virus strains), and different levels of social engagement. Moreover, it does not fully address potential confounding factors, such as socioeconomic status, education level, and access to healthcare, which could influence vaccination rates. Additionally, it did not explore the reasons behind vaccine hesitancy in depth, which would be valuable in understanding the specific concerns of this population.

## 5. Conclusions

Less than half of the pediatric population with CD has been vaccinated against SARS-CoV-2, with young children with CD being exceptionally low; however, it is better in children and adolescents and for all age groups of patients with other associated autoimmune diseases. Vaccine hesitancy remains a concern, particularly in younger age groups and among those diagnosed using the biopsy-sparing approach. Addressing these issues through targeted communication and education efforts is essential to improve vaccination coverage in pediatric patients with CD. Hesitancy increased during the pandemic, requiring a closer collaboration between general pediatricians and specialists in order to improve the effectiveness of vaccination strategies and monitor data on coverage.

Future research should focus on understanding the specific drivers of vaccine hesitancy regarding children with chronic autoimmune diseases and developing interventions to address these concerns. These could include tailored communication campaigns aimed at increasing vaccine confidence and providing clear, evidence-based information about the safety of mRNA vaccines, particularly in children with chronic conditions. Understanding how education level, socioeconomic status, and access to healthcare impact vaccination rates could help identify specific groups that may require additional support. Due to particularly low vaccination rates in children under five years old, research should focus on developing age-appropriate interventions that resonate with parents of younger children. Efforts could focus on dispelling myths surrounding vaccine-related risks and addressing common concerns, such as long-term side effects. In-depth qualitative studies using interviews or focus groups with parents and caregivers could help to uncover specific fears, misconceptions, and barriers to vaccination. Exploring the role of social media and misinformation in influencing vaccine decision-making in these families would also be valuable. Future studies should explore policy-level interventions that could facilitate higher vaccination rates in children with CD. These could include expanding vaccination access through school-based programs, offering flexible vaccination schedules, or implementing policies that encourage collaboration between primary care providers and specialists to ensure vaccination of children with chronic illnesses.

## Data Availability

The data presented in this study are available on request from the corresponding author due to privacy.
